# The effect of thermomechanical welding on the microstructure and mechanical properties of S700MC steel welds

**DOI:** 10.1007/s40194-024-01711-x

**Published:** 2024-02-15

**Authors:** Peng Wang, Felipe Martins Gomes, Fernando Gustavo Warchomicka, Wolfgang Ernst, Rudolf Vallant, Maria Cecilia Poletti, Norbert Enzinger

**Affiliations:** 1 Institute of Materials Science, Joining and Forming at the Graz University of Technology, Kopernikusgasse 24/I, 8010 Graz, Austria; 2 Voestalpine Stahl GmbH, Voestalpine-Strasse 3, 4031 Linz, Austria

**Keywords:** Thermomechanical welding (TMW), Mechanical vibration, S700MC steel, Heat-affected zone softening, Grain refinement

## Abstract

Grain refinement by plastic deformation during conventional TIG welding can help to compensate for the loss of mechanical properties of welded joints. The thermomechanical welding (TMW) tests were performed on S700MC steel with different combinations of TIG arc energy and high frequency hammering over three target cooling times (t_8/5_ = 5s, 15s, and 25s). Additionally, the effect of initial microstructures on the weld joint quality was analysed by testing three materials conditions: hot-rolled (as-received) and cold-rolled with 10% and 30% thickness reductions, respectively. The effects of plastic deformation and the mechanical vibration on the grain refinement were studied separately. Optical microscopy, electron backscattered diffraction, and Vickers hardness were used to characterise the weld microstructure heterogeneity. The weld width and depth and the mean grain size were correlated as the function of cooling time t_8/5_. The results show that the weld dimensions increase with increasing the t_8/5_. The weld microstructures transformed from the mixed martensite and bainite into mixed ferrite and bainite with increasing the t_8/5_ time, and the related mean grain size increased gradually. The TMW welds exhibit smaller grains compared to TIG welds due to the coupled effects of mechanical vibration and plastic deformation. The mechanical vibration contributes to weld metal homogenisation, accelerating TiN precipitation in the fusion zone. The proposed TMW process can refine the weld microstructure of S700MC steel, enhancing its mechanical properties.

## Introduction

High-strength steels (HSSs) are designed for the required strength, achieved by grain refinement, work hardening, solution hardening, precipitation hardening by micro-alloyed elements (Ti, V, and Nb) [1, 2], and phase transformation [3]. Generally, HSSs are produced by reducing the carbon content and adding micro-alloying elements, followed by thermo-mechanical controlled processing (TMCP [4]). TMCP creates a single ferritic microstructure or multi-phase microstructure comprising ferrite and martensite, bainite, or austenite. The cold-formable version of a TMCP HSSs (indicated by MC, with the M referring to the TMCP and the C to the suitability for cold forming [5, 6]) presents the following characteristics: (a) high strength, (b) good formability and (c) good weldability, and (d) high impact resistance. The S700MC steel is an HSS, with a minimum of 700 MPa yield stress and a minimum fracture elongation of 12%. This steel can be used to reduce weight in construction and engineering applications, such as the crane and truck industries [7, 8].

The formation of a soft heat-affected zone (HAZ) is one of the problems after welding S700MC steel [9, 10]. The decrease in the strength of the weld joint can be evaluated by a ratio, viz. the width of the soft zone to the thickness of the material [11, 12]. Reducing the ratio increases the weld joint's strength during tensile tests. According to many researches [13–16], the degree and extent of softening in part of the HAZ are mostly related to the base material, the cooling time *t*_8/5_ [s], and thus the welding procedures [23–26]. The width of the HAZ soft zone expands linearly with an increase in the *t*_8/5_ [17–21]. Usually, a longer heating period above A_1_ temperature leads to more grain growth within the HAZ, decreasing the mechanical properties of the welded joints [14]. The impact of temperature and holding time for S700MC steel on grain growth in the HAZ was investigated by Moravec et al. [22]. The welding CCT (WCCT) diagrams [9, 23, 24] show that the microstructure of S700MC welded joints varies from ‘martensite + bainite’ to ‘bainite + ferrite’ when increasing the cooling time *t*_8/5_ from 5 to 25 s. The martensite block and lath sizes and the martensite fraction and variants influence the mechanical properties of welded joints [25].

Grain refinement is an important method to improve the mechanical properties of a TIG weld. The single plastic deformation through the WeldForming process was used by Adams et al. [26] to improve the weld seam properties of HSSs. Our previous work [27] demonstrated that the plastic deformation combined with frequent vibrations during thermomechanical welding (TMW) can refine the microstructure of the FZ by occurring recrystallisation processes. We noticed that the TMW includes two types of vibrations: mechanical and heat source. Their effects on the refining process are still unclear. Therefore, this work aims to investigate the influence of TMW on the microstructure and mechanical properties of S700MC steel welds, compared with the conventional TIG welding process. The influence of mechanical vibration on the weld joint quality (TIG-V) was primarily evaluated. The obtained microstructures were characterized by optical microscopy (OM) and scanning electron microscopy (SEM) combined with electron backscattered diffraction (EBSD) measurements. The weld dimensions, hardness, and mean grain size of TIG, TMW, and TIG-V welds were correlated to reveal the refinement mechanism of solidified microstructures at various cooling times *t*_8/5_.

## Methodology

### Materials

The investigated S700MC steel was alform 700M plates provided by voestalpine Steel Division. These plates were in three different processing conditions after hot rolling: (i) as-received (AR) condition according to EN 10149–2 [28] with 10-mm thickness, (ii) cold-rolled with 10% (CR10) thickness reduction to 9-mm thickness, and (iii) cold-rolled with 30% (CR30) thickness reduction to 7-mm thickness. Table 1 summarises the chemical composition and Table 2 the typical mechanical properties of the S700MC steel. Generally, the S700MC steel in TMCP conditions derives its desired strength from three hardening mechanisms: grain refinement, precipitation hardening, and a lesser amount of transformation hardening [10, 29].

**Table 1:** Chemical composition of the investigated S700MC steel (in wt%)

Elements	C	Si	Mn	P	S	Al	V + Nb + Ti	Fe
Wt.%	0.06	0.03	1.86	0.009	0.001	0.062	0.195	Balance

**Table 2:** - Mechanical properties and microstructure of the investigated S700MC steel

Processing conditions (thickness)	Main phases	Hardness [HV 10]	*R*_p0.2_ [MPa]	*R*_M_ [MPa]	*R*_p0.2_/*R*_M_
AR (10 mm)	F + B	281	832	868	0.96
CR10 (9 mm)	F + B	305	985	1015	0.97
CR30 (7 mm)	F + B	324	1101	1160	0.95

F and B indicate the ferrite and bainite phases in the S700MC steel, respectively. *R*_p0.2_ [MPa] 0.2% offset yield strength, *R*_M_ [MPa] ultimate tensile strength, *R*_p0.2_/*R*_M_ yield strength ratio

**Fig. 1:**
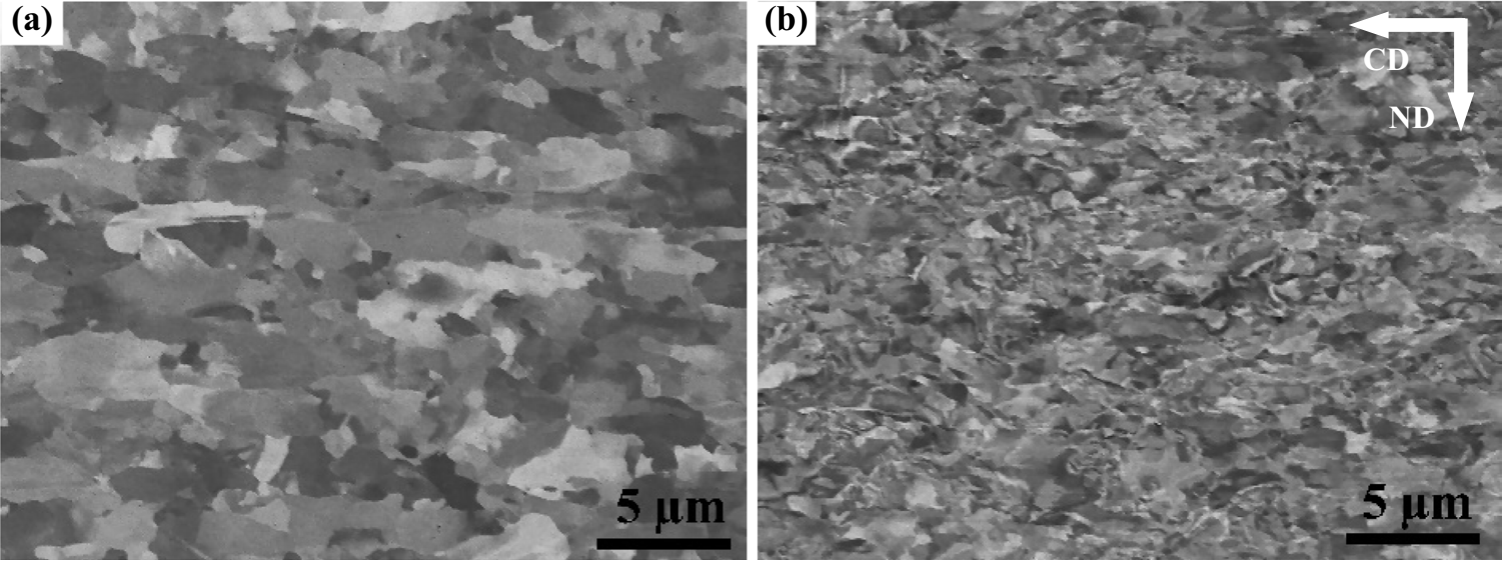
SEM images (BSE mode) of the investigated S700 MC steel at two processing conditions: **a** AR plate and **b** CR30 plate

The S700MC steel mainly contains bainite and ferrite phases [15, 30]. This microstructure provides a good combination of high tensile strength and high fracture toughness. Figure 1a and b show the microstructures of AR-S700MC and CR30-S700MC steel prior to welding, respectively. The cold-rolling process on the AR-S700MC plate modifies the microstructure from nearly equal elongated lamella of ferritic-austenitic grains (Fig. 1a) into largely deformed grains (Fig. 1b). The S700MC steel at the CR30 condition exhibits a larger stored energy, which implies a larger restoration property than that at AR and CR10 conditions during annealing treatment.

### Experimental setup

**Fig. 2:**
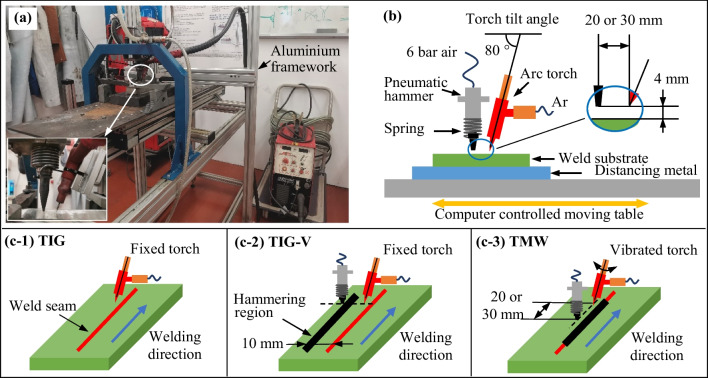
**a** Experimental setup, **b** 2D schematic diagram of the TIG torch and pneumatic hammer position, and **c** the description of three types of welding experiments: (c-1) TIG, (c-2) TIG-V, and (c-3) TMW

Figure 2a presents the experimental setup of the TMW system. The positions of the chisel and torch are shown in Fig. 2b and in our previous works [27, 31, 32]. The arc welding power source was Magic Wave 2000 Fuzzy (produced by Fronius), and welding was with DCEN for all experiments. The shielding gas of Ar (99.9% purity) was applied with a flow rate of 12 l/min. The TIG torch was positioned in two modes: (i) the fixed mode, with the torch held stationary by an aluminium framework (Fig. 2a), and (ii) the vibrated mode according to the TMW process, as described in previous work [31].

In addition, three different bead-on-plate welding modes illustrated in Fig. 2(c-1) to (c-3) describe (c-1): TIG, the conventional tungsten inert gas welding; (c-2) TIG-V, combining the TIG welding with frequency hammering at 35 Hz parallelly to the TIG weld seam by a 10 mm offset; and (c-3) (TMW-*x*), the thermomechanical welding (TMW) with a specific hammering offset (*x* [mm]), followed the TIG welding by frequency hammering using a pneumatic hammer. The pneumatic hammer moves vertically with a free stroke of 13 ± 0.5 mm, and a cycling frequency of 35 Hz at a constant air pressure of 6 bar. Additionally, two kinds of vibrations were acted simultaneously in the TMW process, namely, mechanical and heat-source vibrations [27]. In this work, the hammering offsets of 20 mm and 30 mm were selected to investigate the hammering deformation on modifying the solidified structure at different cooling temperatures. Such two welding experiments are named as TMW-20 and TMW-30, respectively.

### Experimental procedure

The S700MC plates were prepared with identical length and width dimensions (i.e. 100 mm × 50 mm) and tested according to the three bead-on-plate welding types. The welding length of each weld seam is 100 ± 1 mm. Three target cooling times *t*_8/5_ (= 5 s, 15 s, and 25 s) were expected for those welding tests. The welding speed (*v* [mm/min]), and the cooling times t_8/5_ were calculated in the *t*_8/5_ module using the ‘voestapine Welding Calculator’ [33]. Table 3 summarises the calculated *v* [mm/min] and the calculated *t*_8/5_ [s] at specific welding current *I* [A] and welding voltage *U* [V].

**Table 3:** Processing parameters and the calculated cooling time t_8/5_ [s] of welding experiments on the S700MC steel under different welding modes

S700MC steel	Welding types	Target cooling time *t*_*8/5*_ [s]											
		5.0				15.0				25.0			
		*v* [mm/min]	*U* [V]	*I* [A]	*t*_*8/5*_ [s]	*v* [mm/min]	*U* [V]	*I* [A]	*t*_*8/5*_ [s]	*v* [mm/min]	*U* [V]	*I* [A]	*t*_*8/5*_ [s]
AR (10 mm)	TIG	103	13.3	125	4.5	-	-	-	-	46	13.1	125	21.9
		154	14.1	175	4.4	98	17.3	175	16.5	69	13.9	175	21.5
	TIG-V	103	13.0	125	4.3	-	-	-	-	46	12.5	125	19.9
		154	13.8	175	4.2	98	15.1	175	12.6	69	13.5	175	20.3
	TMW-20	103	14.0	125	5.0	-	-	-	-	46	13.9	125	24.6
		154	13.7	175	4.2	90	15.2	175	15.1	69	12.9	175	18.5
	TMW-30	103	12.4	125	4.0	-	-	-	-	46	12.0	125	18.4
		154	13.2	175	3.9	-	-	-	-	69	13.1	175	19.1
CR10 (9 mm)	TIG	179	15.1	175	4.6	112	15.0	175	14.4	80	14.9	175	22.5
	TIG-V	179	13.7	175	3.8	112	15.2	175	12.0	80	13.4	175	18.2
	TMW-20	179	13.2	175	3.5	112	15.0	175	11.7	80	13.0	175	17.1
	TMW-30	179	13.7	175	3.8	-	-	-	-	80	13.5	175	18.5
CR30 (7 mm)	TIG	257	18.2	175	5.4	149	18.5	175	16.7	115	18.0	175	26.5
	TIG-V	257	15.2	175	3.8	149	15.6	175	11.8	115	15.4	175	19.4
	TMW-20	257	15.8	175	4.1	149	15.2	175	11.2	115	15.5	175	19.6

“-” indicates there are no welding tests at such conditions. *v* the welding speed [mm/min], *U* welding voltage [V], *I* welding current [A] of a TIG welding. AR denotes the as-received plate, and CR10 and CR30 are cold-rolled plates by the 10% and 30% thickness reduction of AR-S700MC steel in delivery condition, respectively

**Fig. 3:**
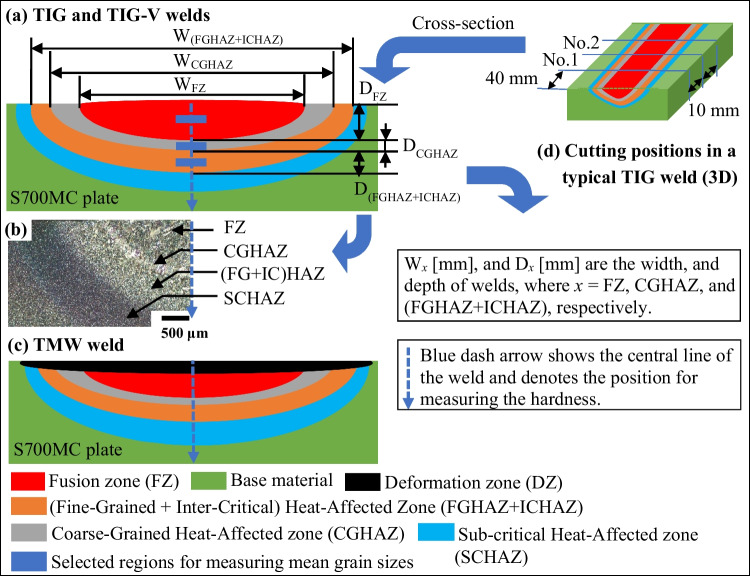
The 2D schematic diagrams of a TIG bead-on-plate weld (**a**), a physical semi-TIG weld (**b**), a TMW weld (**c**), and the positions (**d**) (No.1 and No.2) for cutting the metallography samples in a typical TIG weld

Five distinct regions at the cross-section of a typical TIG weld were identified: (i) the fusion zone (FZ); (ii) the coarse-grained heat-affected zone (CGHAZ); (iii) the region composed of two sub heat-affected zones (FGHAZ + ICHAZ) [34], i.e. fine-grained heat-affected zone (FGHAZ, *T* > A_3_) and inter-critical heat-affected zone (ICHAZ, A_1_ < *T* < A_3_); (iv) the sub-critical heat-affected zone (SCHAZ [35], *T* < A_1_); and (v) the base material (BM). Figure 3a shows these five regions schematically and verified experimentally as shown in Fig. 3b. In addition, the deformation zone (DZ) in black appears in the TMW weld due to the frequency hammering (see Fig. 3c). The geometry and size of the DZ is highly dependent on the chisel tip dimension [27]. Figure 3a shows the dimensions of both TIG and TMW welds, i.e. the width (W_*x*_) and depth (D_*x*_), where *x* = FZ, CGHAZ, and (FGHAZ + ICHAZ).

The standard metallographic techniques were used to analyse the microstructures of performed samples. Two samples were extracted from each weld to evaluate the microstructure at the cross-section, and their positions for cutting and observing (i.e. No.1 and No.2 cross-sections) are shown in Fig. 3d. These samples were etched with Crida QP plus solution for 15 min. The microstructures were captured using an optical microscope (Zeiss Observer Z1m with an Axio-Cam-MRC5 camera) and also a field emission gun (FEG)-Scanning Electron Microscopy (TESCAN Mira3) equipped with an EDAX-Hikari camera for Electron Backscattered Diffraction (EBSD) measurements. EBSD measurements in selected regions had an area of (150 × 150 mm^2^), scanned with a step size of 0.1 at 25 kV and beam size of about 30 nm. The camera (Nikon-N90) was used to obtain the macro images of welds and the geometry of the FZ, CGHAZ, and (FGHAZ + ICHAG), i.e. width (W_*x*_) and depth (D_*x*_) as depicted in Fig. 3a. Moreover, at least 5 LOM images were captured from the blue regions of each zone to determine the mean grain size. The prior austenite grain size (PAGS) of FZ and CGHAZ, and final grain size of (FGHAZ + ICHAZ) were measured using the line intersection method according to ISO 643:2019 standard. The related data was processed by ImageJ and Gimp [36] and analysed using the MATLAB code programmed by Lehto et al. [37]. The hardness distribution over the weld cross-section was determined according to the Vickers (HV 0.5) measurement method using the DuraScan G5 (0.00025–62.5 kgf) [38]. The hardness distributions over a line and a rectangular region were determined. The line measurement is vertical from the weld top surface to the base material, as indicated by dashed blue arrows in Fig. 3a and c, and the mapping measurement covers the full weld by a rectangular area of 9 mm × 6.6 mm.

## Results

### Weld dimensions

**Fig. 4:**
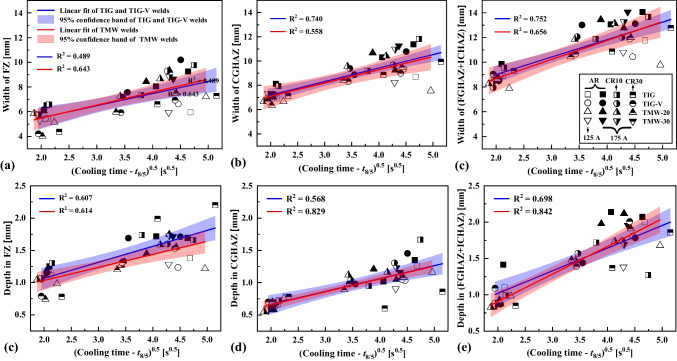
Dimensions of S700MC steel welds as function of the cooling time t_8/5_, where (**a**) ~ (**c**) the depth and (**d**) ~ (**f**) the width of different weld zones

Figure 4 shows the distribution of dimensions of S700MC welds as a function of the cooling time *t*_8/5_. The distributions are in the target cooling time range of [5 s, 25 s]. The weld width (see Fig. 4a until c) and depth (see Fig. 4e until f) of FZ, CGHAZ, and (FGHAZ + ICHAZ) increase with increasing *t*_8/5_. Generally, the depth of CGHAZ is relatively smaller than that of FZ and (FGHAZ + ICHAZ) at a given *t*_8/5_. The linear fitting technique was applied to reveal the relationship between weld dimensions and the square root of *t*_8/5_. All data points were categorised into two sets: (i) TIG and TIG-V welds and (ii) TMW welds, including TMW-20 and TMW-30 welds. According to Eq. 1, the linear fitting models based on such two data sets were obtained using the least-square regression method, and those fitting coefficients of ${{\text{a}}}_{x,y}$ [mm/s^0.5^] and ${b}_{x,y}$ [mm] (*x* = depth or width and *y* = FZ, CGHAZ, and (FGHAZ + ICHAZ)) are summarized in Table 4 as shown in Appendix 1.

1 $$\begin{aligned}D_{x,y}&=a_{x,y}\bullet\sqrt{t_{8/5}}+b_{x,y}(x=\mathrm{depth\;or\;width},\,\\&y=\text{FZ},\,\text{CGHAZ}\;\text{or}(\text{FGHAZ}+\text{ICHAZ}))\end{aligned}$$

Figure 4 includes the experimental points, all fitting curves, and the 95% confidence bands. The depth and width of all welds continuously increased over the cooling time *t*_8/5_. The 95% confidence band represents considerable uncertainty at a larger *t*_8/5_, especially near the *t*_8/5_ of 25 s due to the larger heat dissipation. Furthermore, the TMW process broadens the FZ width as the result of flattening influence due to the plastic deformation, especially in the *t*_8/5_ range of [5 s, 20 s] (see Fig. 4d), which is similar to the work on the austenitic stainless steel [27].

The ${t}_{8/5}$ in Eq. 1 can be described by Eq. 3 (seen in Appendix 2), or Eq. 4 by considering the welding parameters and material properties. In Eq. 2, the $U$, $I$, and $v$ are the typical bead-on-plate TIG welding parameters, i.e. the welding voltage [V], welding current [A], and welding speed of the TIG torch [mm/s]. JMatPro 13.3 software was used to obtain the material constants as functions of the temperature [K], $\lambda$ thermal conductivity [J/(cm⋅K⋅s)], $\varrho$ mass density [g/cm^3^], and $c$ specific heat capacity [J/(g⋅K)]. They were modelled polynomially by the least-square regression method, and plotted in Fig. 15 (Appendix 2). In the present study, $\lambda$, $\varrho$, and $c$ are assigned values of 0.334, 7.61, and 0.9, which were calculated at 650 °C from those fitting curves, respectively.

2 $${t}_{8/5}=\frac{a}{\lambda \varrho c}\bullet {\left(\frac{\eta UI}{vd}\right)}^{2}\bullet \left[{\left(\frac{1}{500-{T}_{0}}\right)}^{2}-{\left(\frac{1}{800-{T}_{0}}\right)}^{2}\right]+b$$

${T}_{0}$ is the preheating temperature [°C] and is equal to 20 °C in the work, $\eta$ is the thermal efficiency with an empirical value of 0.65 selected for the bead-on-plate TIG welding process (DCEN), and $d$ is the workpiece thickness [mm]. Moreover, *a* and *b* in Eq. (2) are two fitting parameters, evaluated as 0.30 and 0.042, respectively.

### Hardness

**Fig. 5:**
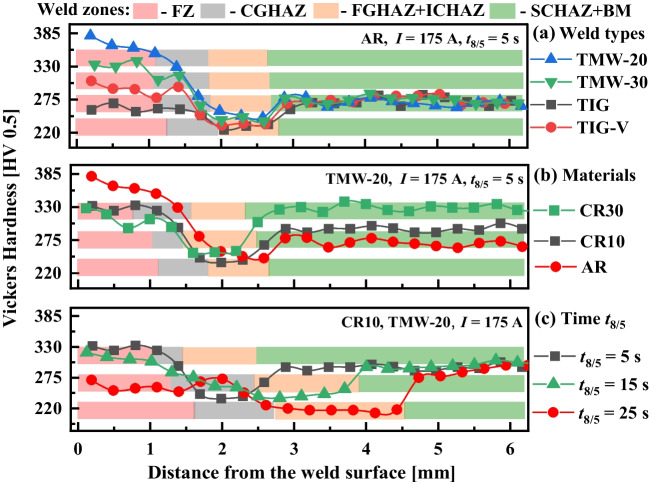
Line hardness distribution along a vertical line of S700MC steel welds at different welding conditions: **a** four welding types (i.e. TIG, TIG-V, TMW-20, and TMW-30), **b** three different substrates (i.e. AR, CR10, and CR30-S700MC steel plates), and **c** three target cooling times t_8/5_ (5 s, 15 s, and 25 s)

Figure 5 shows the line hardness (HV0.5) distributions along the central line of the weld top surface to the base material (as illustrated in Fig. 3a and c) for different conditions. Each hardness profile shows higher hardness in the FZ and DZ at the surface. Then, the hardness decreases gradually in the CGHAZ until the minimum in the (FGHAZ + ICHAZ) zones. Finally, the hardness increases and stabilises in the base material. In addition, Fig. 5 shows the hardness along the different welding zones as a function of the welding type, the condition of the base material, and the target cooling time *t*_8/5_.

Figure 5a shows the hardness distributions for different welding types at a welding current of 175 A and an identical target cooling time *t*_8/5_ of 5 s. The highest hardness obtained on the TMW weld is a consequence of the work hardening and mechanical vibration. The specific hammering offset also influences the hardness significantly; larger hardness occurs in the TMW-20 weld compared with the TMW-30 weld. In addition, the single mechanical vibration significantly enhances the weld hardness of the TIG-V weld compared to the conventional TIG weld. Figure 5b shows the influence of the initial material on the weld hardness of TMW-20 welds. As expected, the hardness of the base material is the largest in CR30 due to cold working. Furthermore, the hardness over FZ is different for all the materials because the calculated cooling time *t*_8/5_ is different due to the thicknesses of the investigated plates. Finally, Fig. 5c demonstrates the significant effect of *t*_8/5_ on the weld hardness. As increasing the target cooling time *t*_8/5_, the hardness in the FZ decreases. Meanwhile, the weld dimensions enlarge gradually with an increasing softening feature, especially for the (FGHAZ + ICHAZ).

### Microstructures of different welds

#### Microstructures of the TIG and TIG-V welds

**Fig. 6:**
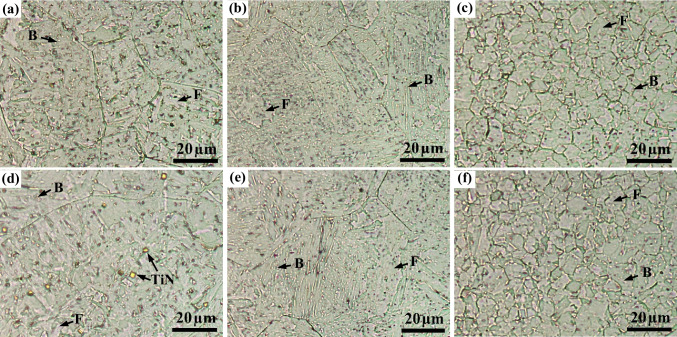
LOM images of the AR-S700MC steel welds on different weld zones: **a** and **d** FZ, **b** and **e** CGHAZ, and **c** and **f** FGHAZ (nearby the CGHAZ boundary) of TIG (**a–c**) and TIG-V (**d–f**) welds obtained at an identical welding current of 175 A and target cooling time t_8/5_ of 15 s

Figure 6 shows the microstructures of different weld zones (i.e. FZ, CGHAZ, and (FGHAZ + ICHAZ)) of TIG, and TIG-V welds, carried out on the AR—S700MC steel at the welding current of 175 A and target cooling time *t*_8/5_ of 15 s. A significant microstructure heterogeneity was observed among the three weld zones. The FZ and CGHAZ exhibit larger grains than (FGHAZ + ICHAZ). The welds are mainly composed of two different microstructures as indicated by black arrows: a ferrite (F) and b bainite (B), as found in previous studies [23, 24]. Some carbides and TiN [2, 23, 34] appear inside the FZ grains of the TIG-V welds (Fig. 6d) compared to the TIG welds (Fig. 6a), revealing that the mechanical vibration promotes the formation of precipitates. However, the TIG and TIG-V welds have no significant differences in the microstructures between CGHAZ and (FGHAZ + ICHAZ).

#### Microstructures of the TMW welds

**Fig. 7:**
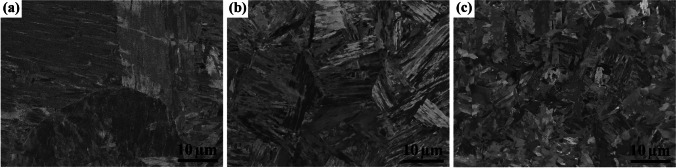
SEM images (BSE mode) of AR-S700MC steel weld on different zones: **a** FZ, **b** CGHAZ, and **c** (FGHAZ + ICHAZ) of the TMW-20 weld obtained at the welding current of 175 A and target cooling time t_8/5_ of 5 s

Figure 7 shows the microstructures of a typical TMW-20 weld performed on the AR-S700MC steel plate at the welding current of 175 A and target cooling time *t*_8/5_ of 5 s. It can be seen in Fig. 7a that the FZ presents the largest prior austenite grains and thinnest martensite laths due to the high solidification cooling rate. Figure 7b shows the CGHAZ exhibits wider martensite laths and larger martensite blocks than the FZ. Finally, in Fig. 7c, the (FGHAZ + ICHAZ) has a fully α-ferrite microstructure instead of forming martensite.

**Fig. 8:**
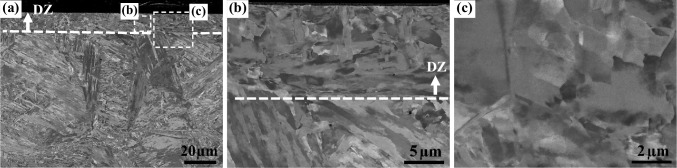
SEM images (BSE mode) of the deformation zone of AR-S700MC steel weld obtained by a TMW-20 test at welding current of 175 A and cooling time t_8/5_ of 5 s, which observe different magnifications of BSE images: **a** low magnification and **b**, **c** high magnifications of rectangular regions marked in (**a**), respectively

The deformation zone (DZ, in Fig. 3b) microstructures of this TMW-20 weld is displayed in Fig. 8 with three different magnifications. Figure 8 a and b show that the DZ region is of about 10 μm depth, indicated by the white arrows above the dash lines. Figure 8b shows the largely deformed structures in DZ, but also the fine microstructures in Fig. 8c is observed, related to dynamic recrystallisation with small recrystallised grains and/or substructures. The region below the dashed line belongs to FZ, characterised by big prior austenite grains and thin lath-martensite due to the high cooling rate.

### Grain size

**Fig. 9:**
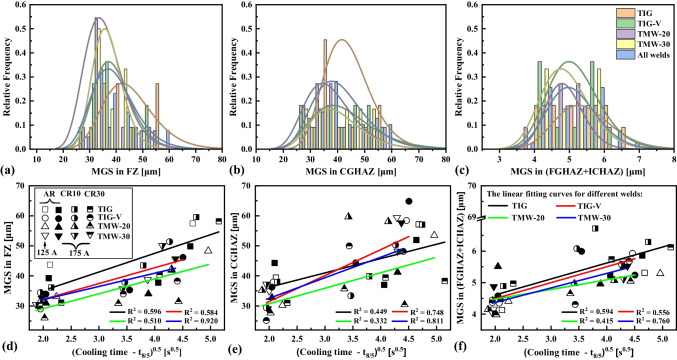
Distribution of the mean grain size (MGS) versus the cooling time t_8/5_ and the associated histogram with distribution curves (lognormal type) for different weld zones of S700MC steel: **a**, **d** FZ, **b**, **e** CGHAZ, and **c**, **f** (FGHAZ + ICHAZ) at different welding conditions

Figure 9 shows the mean gain size (MGS) distribution of different weld zones (i.e. FZ, CGHAZ, and (FGHAZ + ICHAZ)) with respect to the square root of *t*_8/5_ of S700MC steel over the TIG, TIG-V, TMW-20, and TMW-30 welding types. It indicates that the MGS of these three weld zones increases as increasing the target cooling time *t*_8/5_ from 5 to 25 s [22].For simplicity, the MGS for each weld zone was quantified by considering all these four welding types, and as increasing the target *t*_8/5_ from 5 to 15 s and further to 25 s, the MGS were listed as follows: (i) 31.09 ± 3.36 μm, 38.37 ± 5.18 μm, and 47.70 ± 8.24 μm for the FZ; (ii) 33.69 ± 5.24 μm, 41.92 ± 7.64 μm, and 50.98 ± 7.75 μm for the CGHAZ; and (iii) 4.49 ± 0.42 μm, 5.40 ± 0.63 μm, and 5.63 ± 0.41 μm for the (FGHAZ + ICHAZ). Figure 9a, b, and c display the histogram distributions of MGS on the FZ (without considering the DZ), CGHAZ, and (FGHAZ + ICHAZ), and the associated linear fitting curves of MGS are plotted in Fig. 9d, e, and f, respectively. The fitting coefficients are listed in Table 6.

Figure 9a and d show that in the FZ, the TIG method produces a larger MGS of 45.16 ± 9.73 μm than TIG-V welds with the MGS of 38.94 ± 7.48 μm, and TMW-20 welds exhibit the smallest MGS of 36.09 ± 6.89 μm. The mechanical vibration process relatively refines the MGS of FZ, and the TMW process further enhances the grain refinement by the coupled influence of mechanical vibration and plastic deformation on the weld metal. However, the MGS of CGHAZ becomes larger for the TIG-V welds than for TIG welds when the cooling time *t*_8/5_ is increased, as observed in Fig. 9b and e. In addition, the MGS of (FGHAZ + ICHAZ) is smaller for TMW welds than for TIG welds (Fig. 9c and f), probably attributed to the higher heat dissipation from the frequent hammering.

## Discussion

The microstructure of S700MC welds is sensitive to the cooling rate (or the cooling time *t*_8/5_), the mechanical vibration, and the plastic deformation.

### Effect of the cooling rate on the weld microstructure evolution

**Fig. 10:**
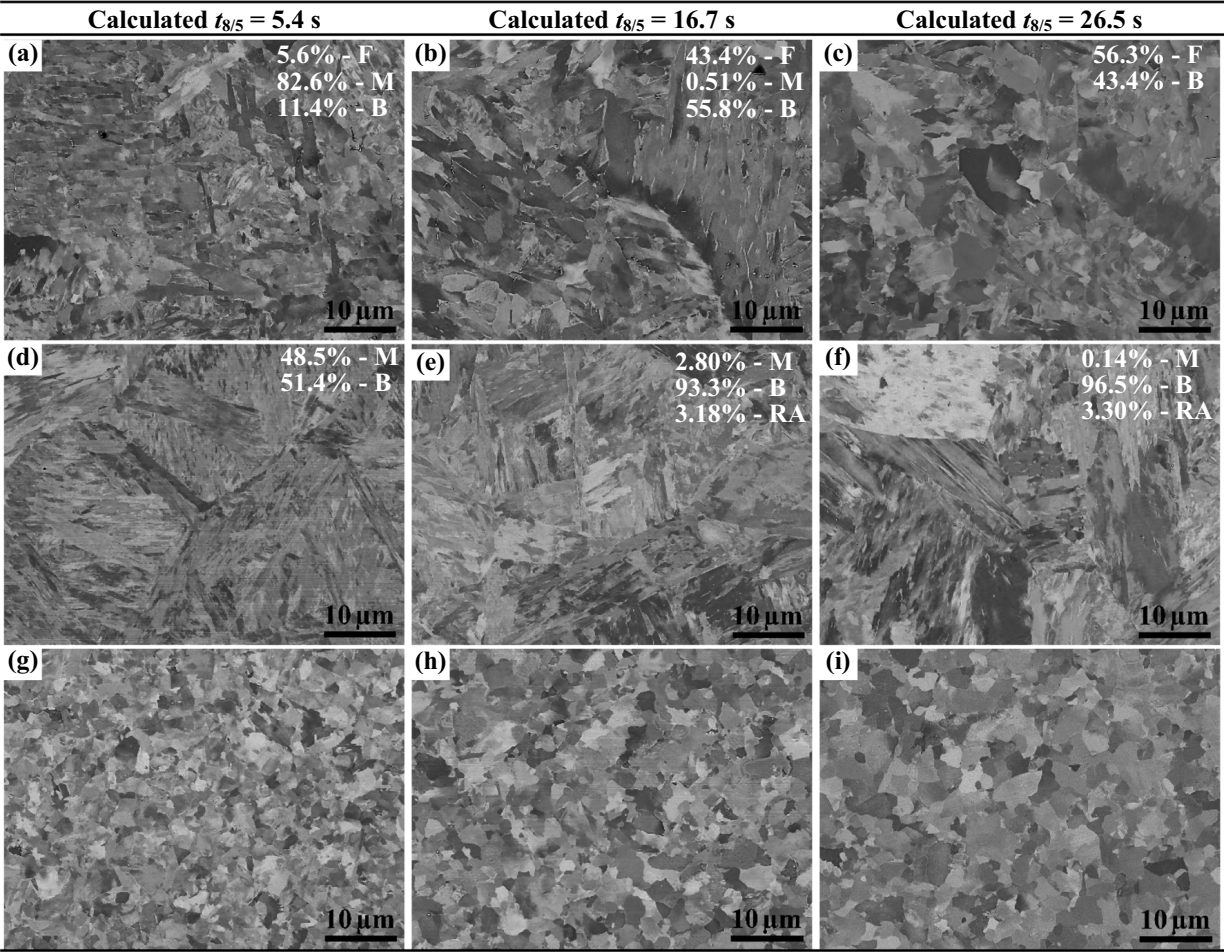
SEM images (BSE mode) of TIG welds: **a**, **b**, **c** FZ, **d**, **e**, **f** CGHAZ, **g**, **h**, **i** the (FGHAZ + ICHAZ) of CR30-S700MC steel welds at three different cooling times t_8/5_

Figure 10 displays the BSE images of different weld zones of unetched TIG welds, performed on the CR30-S700MC steel at the welding current of 175 A over three calculated cooling times *t*_8/5_ of 5.4 s, 16.7 s and 26.5 s (see Table 3). The related HAZ microstructures were calculated according to the fitted models (at the peak *T* = 1300 °C) as shown in Fig. 16 (Appendix 3). Moreover, the evolution of FZ microstructures at the calculated *t*_8/5_ times were calculated using the JMatPro 13.3 at the given PAGS and plotted in Fig. 17 (Appendix 3). The corresponding microstructure fractions are marked in Fig. 10.

Figure 10 a, b, and c show the microstructures of FZ at calculated *t*_8/5_ times of 5.4 s, 16.7 s, and 26.5 s, respectively. Figure 10a shows that a large amount of martensite (~ 82.6% calculated in Fig. 17a) forms together with bainite and ferrite (i.e. ‘B + M + F’). Furthermore, the martensite lath is characterised by a thin width. As increasing the *t*_8/5_ into 16.7 s, there is still a tiny portion of martensite observed in the FZ, as shown in Fig. 10b, and the FZ mainly includes ferrite and bainite (i.e. ‘F + B’). At the longest cooling time of 26.5 s, the FZ entirely composes of ferrite grains mixed with bainite microstructure (i.e. ‘F + B’) as shown in Fig. 10c, similar to Fig. 6a. Overall, it is concluded that the shorter the cooling time *t*_8/5_ is, the more martensite forms in the FZ. Figure 10 d, e, and f show the microstructures of CGHAZ at calculated *t*_8/5_ of 5.4 s, 16.7 s, and 26.5 s, respectively. It is observed that the martensite in the CGHAZ becomes less and less as increasing the *t*_8/5_. Finally, the (FGHAZ + ICHAZ) microstructures shown in Fig. 10 g, h, and i present ferrite grains that become larger and larger as increasing the calculated *t*_8/5_ from 5.4 s to 26.5 s due to the intensive restoration processes.

### Effect of the single mechanical vibration on the weld metal refinement

**Fig. 11:**
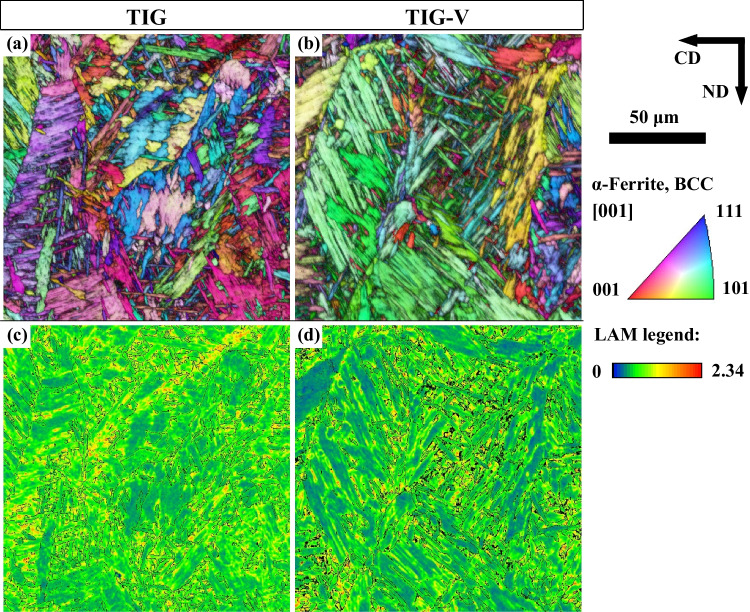
EBSD maps of the α-ferrite phase for TIG and TIG-V welds on AR—S700MC steel at the welding current of 175 A and target cooling time t_8/5_ of 5 s: **a**, **b** overlapping the inverse pole figure (IPF) colour map and image quality (IQ) map and **c**, **d** local average misorientation (LAM) colour map. Black and white lines in the LAM map indicate the HAGB and LAGB, respectively

Figure 11 shows the inverse pole figure (IPF) overlapping the image quality (IQ) map and the local average misorientation map (LAM) of α-ferrite in the FZ of TIG and TIG-V welds, performed on the AR-S700MC steel at 175 A and target cooling time *t*_8/5_ of 5 s. Black and white lines in LAM maps refer to high-angle grain boundary (HAGB) or interfaces and low-angle grain boundary (LAGB), respectively. Figure 11a and b reveal that martensite formed in the FZ. The LAM maps show the TIG-V weld with less misorientation and less number of secondary dendrites [39] than TIG weld, probably ascribed to the positive effect of homogenisation and formation of martensite in the FZ through the mechanical vibration during the solidification stage.

**Fig. 12:**
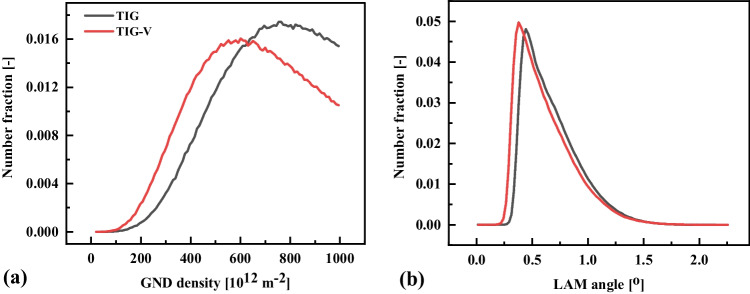
EBSD charts of geometry necessary dislocation (GND) density and local average misorientation (LAM) of TIG and TIG-V welds as shown in Fig. 11 on the AR-S700MC steel at the welding current of 175 A and target cooling time t_8/5_ of 5 s

Figure 12 shows the comparative analysis of the geometry necessary dislocation (GND) density [m^−2^], and local average misorientation (LAM) of Fig. 11. It is observed that the peak value of GND and LAM distribution curves is lower for the TIG weld than TIG-V weld. It reveals that the single mechanical vibration decreases the local misorientation gradient of the FZ solidified microstructure.

### Effect of thermomechanical hammering on the grain refinement

**Fig. 13:**
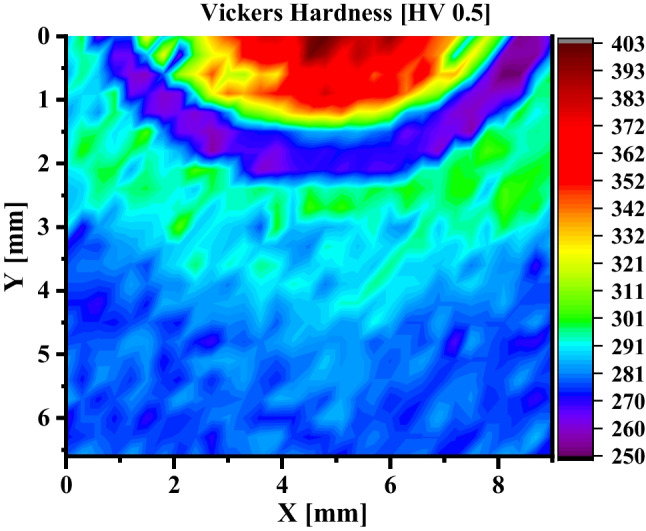
The Vickers hardness (HV 0.5) map over a TMW-20 weld of AR-S700MC steel at the welding current of 175A and target cooling time t_8/5_ of 5 s

Figure 13 shows the hardness map (HV 0.5) over the cross-section of a TMW-20 weld of AR-S700MC steel at the welding current of 175A and target cooling time of 5 s. The hardness map shows five specific weld zones as depicted in Fig. 3 by different colours: (i) red, FZ; (ii) purple, (FGHAZ + ICHAZ); (iii) the green between red and purple is the CGHAZ; (iv) the green between purple and blue is the sub-critical HAZ (SCHAZ, T < A_1_); and (v) the blue region represents the BM. In addition, the deep red coloured region in the top region of FZ represents the DZ (as illustrated in Fig. 3c) with the highest hardness (~ 400 HV 0.5). The FGHAZ + ICHAZ exhibits the smallest hardness of around 260 (HV 0.5) following the observations in Fig. 5a. The hardness distribution correlates to the microstructure heterogeneity of the TMW weld.

**Fig. 14:**
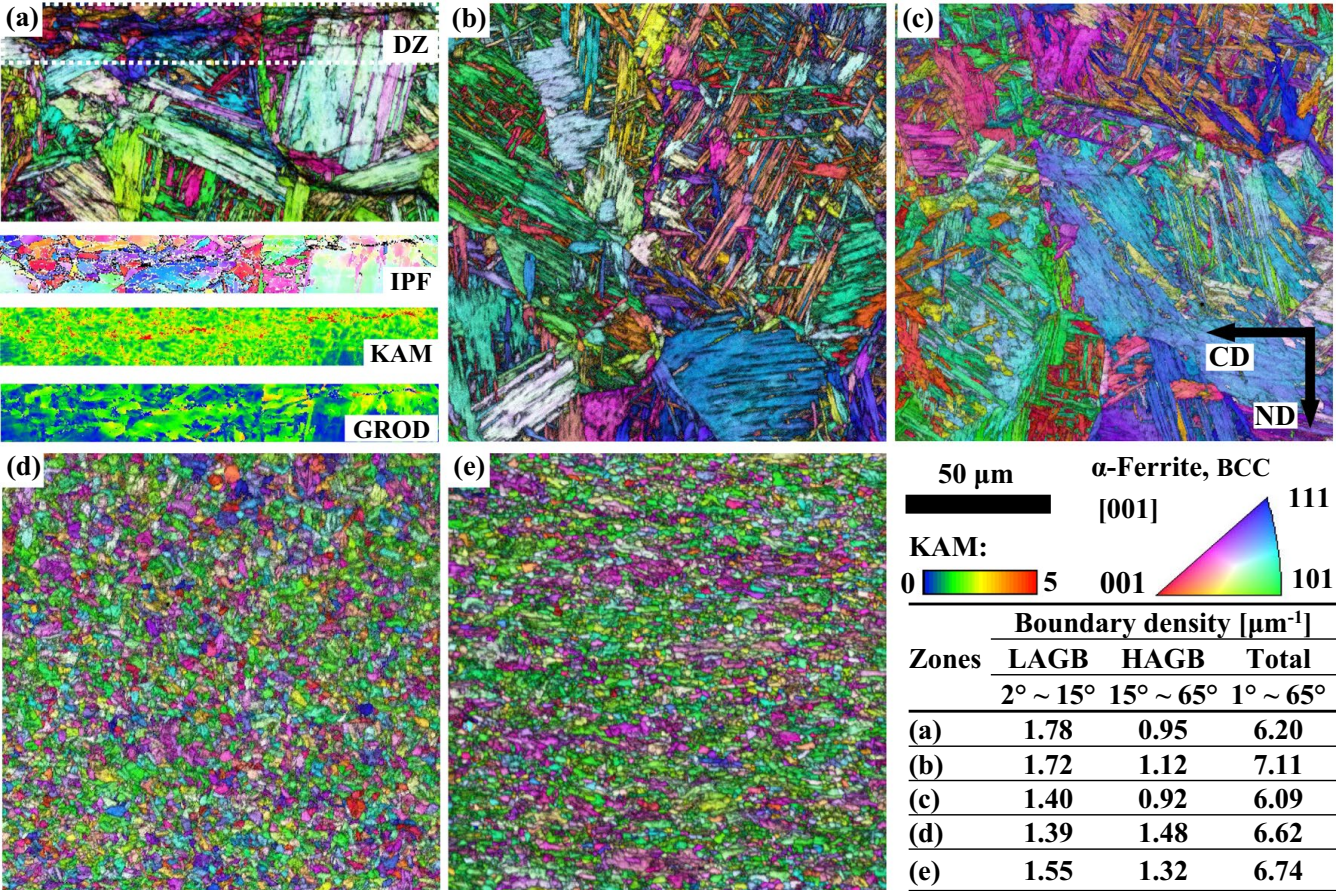
EBSD maps. Inverse pole figure (IPF) overlapping the image quality (IQ) map of α-ferrite for different weld zones: **a** DZ, **b** FZ, **c** CGHAZ, **d** (FGHAZ + ICHAZ), and **e** BM of a TMW-20 weld on AR—S700MC steel at the welding current of 175 A and target cooling time t_8/5_ of 5 s. The single IPF, Kernel average misorientation (KAM), and Grain reference orientation deviation–angle (GROD-angle) maps of DZ marked by the rectangular dashed line are shown in (**a**)

**Fig. 15:**
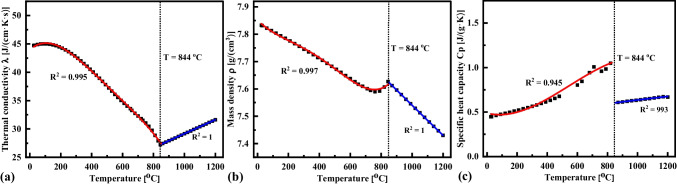
Plots of thermal conductivity (**a**), mass density (**b**), and specific heat capacity (**c**) of the S700MC steel respective to temperature [°C] and the associated polynomial fitting curves using a least-square fitting method

**Fig. 16:**
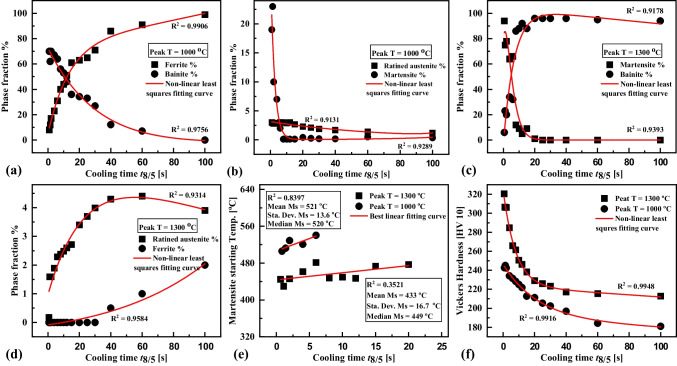
Compositions of the S700MC steel (**a** ~ **d**), martensite starting temperature (Ms [°C]) (**e**), and Vickers hardness (HV 10) (**f**) versus the cooling time t_8/5_ [s] for two peak heating temperatures of 1000 °C at the heating rate of 340 °C/s and 1300 °C at the heating rate of 575 °C/s, and the associated fitting curves

**Fig. 17:**
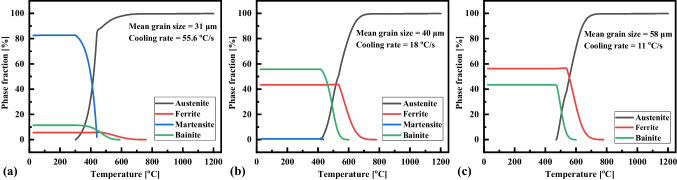
Diagrams of microstructure fraction for S700MC steel with respect to the temperature [°C] at different cooling rates

Generally, the TMW process provides plastic deformation and mechanical vibration on the weld metal. Figure 14 shows the inverse pole figure (IPF) and image quality (IQ) maps of the α-ferrite phase for five typical regions (i.e. DZ, FZ, CGHAZ, (FGHAZ + ICHAZ) and BM in a TMW-20 weld. The boundary densities [μm^−1^], categorised into ranges of LAGB (2°–15°), HAGB (15°–65°), and a broader range (1°–65°) and calculated from EBSD results and displayed in the table presented in Fig. 14. A highly deformed microstructure of DZ was observed as shown in Fig. 14a with high KAM values. Figure 14b and c display the characteristic hierarchical structure of lath-martensite in FZ and CGHAZ, respectively. It shows that more lath-martensite formed in the FZ compared to the CGHAZ. The more fraction of martensite is directly related to the higher hardness in FZ compared to CGHAZ [13, 14]. Figure 14d shows the microstructure of FGHAZ without lath-martensite but with refined and equiaxed α-ferrite ascribed to the high-temperature cycling influence. The static recrystallisation of the rolled base material occurred in the FGHAZ (after comparison to the rolled microstructures in Fig. 14e). In a word, the TMW process exhibits two main benefits in modifying the coarse microstructure of TIG welds: (i) the mechanical vibration for promoting the precipitation (e.g. the TiN precipitate in S700MC steel weld) in the FZ and homogenisation of solidified microstructure by decreasing the local average misorientation and (ii) the plastic deformation for refining the deformed microstructure through recrystallisation process, and thus increasing the hardness contributed by work hardening.

### Summary and conclusions

In this work, thermomechanical welding (TMW)and two TIG welding tests were performed on the S700MC steel at different welding conditions to find a correlation between microstructure, processing, and mechanical properties. The investigated materials are three kinds of plates after hot rolling (AR—hot-rolled), cold rolling with 10% (CR10), and cold rolling with 30% (CR30) of thickness reductions. Bead-on-plate TIG welding tests were carried out over three target cooling times *t*_8/5_ (i.e. 5 s, 15 s, 25 s) at welding currents of 125 A and 175A. The three types of welding tests are (i) the conventional TIG welding, (ii) the TIG welding with fixed TIG torch and frequent hammering near the weld seam with 10 mm offset (i.e. TIG-V), and (iii) the regular TMW with 20 mm and 30 mm hammering offset (TMW-20 and TMW-30). The influence of single mechanical vibration on the weld mechanical properties and microstructure was evaluated. Several conclusions are as follows:

1. The S700MC steel welds present large microstructure heterogeneity, mainly composed of FZ, CGHAZ, and (FGHAZ + ICHAZ) for the TIG and TIG-V welds, and one more zone, i.e. the deformation zone (DZ) with largely deformed microstructures in TMW welds. The CGHAZ and (FGHAZ + ICHAZ) have lower Vickers hardness distribution.
2. The mean grain size (MGS) of FZ, CGHAZ, and (FGHAZ + ICHAZ) of S700MC steel welds were evaluated at different target *t*_8/5_: (i) 31.09 ± 3.36 μm, 33.69 ± 5.24 μm, and 4.49 ± 0.42 μm at target *t*_8/5_ = 5 s, (ii) 38.37 ± 5.18 μm, 41.92 ± 7.64 μm, and 5.40 ± 0.63 μm at target *t*_8/5_ = 15 s, and (iii) 47.70 ± 8.24 μm, 50.98 ± 7.75 μm, and 5.63 ± 0.41 μm at target *t*_8/5_ = 25 s. The related MGS were linearly modelled as a function of the square root of *t*_8/5_. The fitted models indicate that the MGS of all weld zones increases as increasing the target *t*_8/5_ from 5 to 25 s. As increasing the *t*_8/5_, the weld microstructures were transformed from mixed martensite and bainite (i.e. M + B) into the mixed ferrite and bainite (i.e. F + B), and the lathy martensite becomes less amount but thicker in width.
3. The single mechanical vibration can contribute to the homogenisation of local microstructure, and accelerate the TiN precipitation in FZ. Moreover, the coupled influences of mechanical vibration and plastic deformation of TMW generally promote the hardness and microstructure refinement of S700MC steel welds, enhancing the mechanical properties of TMW welds.

## Appendix 1

Dimensions S700MC steel welds include two components: (a) width and (b) depth of the fusion zone (FZ), coarse-grained heat-affected zone (CGHAZ), and fine-grained and inter-critical heat-affected zone (FGHAZ + ICHAZ). Dimensions of such three weld zones are subjected to the linear fitting technique to the square root of *t*_8/5_ to obtain regression models. The corresponding coefficients of fitted models for both TIG and TIG-V welds are summarised in Table

**Table 4:** Coefficients of the linear fitting models for describing the function of TIG and TIG-V weld dimensions of the S700MC steel versus the square root of cooling time t_8/5_ [s]

Dimensions of S700MC steel weld zones	Coefficients of the linear fitting models for TIG and TIG-V welds					
	FZ		CGHAZ		FGHAZ + ICHAZ	
	a [mm/s^0.5^]	b [mm]	a [mm/s^0.5^]	b [mm]	a [mm/s^0.5^]	b [mm]
Depth [mm]	0.24769	0.57234	0.20055	0.26212	0.31227	0.39434
Width [mm]	0.97978	3.54334	1.11594	4.95561	1.41409	6.1764

^4^.

For thermomechanical welding (TMW) of metals, frequent hammering broadens the weld metal dimensions, precisely the width of FZ. Thus, the TMW weld dimensions were modelled separately from the (TIG and TIG-V) welds. Here, the associated coefficients of linear fitting models (i.e. Equation 1) are summarised in Table

**Table 5:** Coefficients of the linear fitting models describing the function of TMW weld dimensions of S700MC steel versus the square root of cooling time t_8/5_ [s]

Dimensions of S700MC steel weld zones	Coefficients of the linear fitting models for TMW-20 and TMW-30 welds					
	FZ		CGHAZ		FGHAZ + ICHAZ	
	a [mm/s^0.5^]	b [mm]	a [mm/s^0.5^]	b [mm]	a [mm/s^0.5^]	b [mm]
Depth [mm]	0.20193	0.63118	0.20009	0.25761	0.38062	0.14434
Width [mm]	1.04977	3.37943	1.09098	4.91272	1.53642	5.61668

^5^.

Similarly, by using the least-square fitting method, the mean grain size of these three weld zones (i.e. FZ, CGHAZ, and (FGHAZ + ICHAZ)) was linearly fitted as a function of the square root of *t*_8/5_, (see Eq. 1). The related coefficients are summarised in Table

**Table 6:** Coefficients of the linear fitting models describing the function of mean grain size versus the square root of cooling time t_8/5_ [s] for different weld zones of S700MC steel

Different welding types	Coefficients of the linear fitting models for mean grain size					
	FZ		CGHAZ		FGHAZ + ICHAZ	
	a [μm/s^0.5^]	b [μm]	a [μm/s^0.5^]	b [μm]	a [μm/s^0.5^]	b [μm]
TIG + TIG-V	6.08331	21.04931	6.43971	21.02442	0.51411	3.51160
TMW	4.63761	21.06033	5.60401	20.24599	0.35610	3.71648
TIG	6.20861	22.67707	4.76618	26.69747	0.50622	3.59753
TIG-V	5.26825	21.64319	8.64005	14.18970	0.50573	3.48176
TMW-20	4.95766	19.17947	5.16184	20.39903	0.30662	3.86261
TMW-30	4.40916	23.25642	6.83803	18.86840	0.46127	3.44839

^6^, and separated by TMW and (TIG + TIG-V) weld groups as well.

## Appendix 2

The calculation of cooling time *t*_8/5_ is simplified according to EN 1011–2. When welding products of relatively small thickness, two-dimensional heat dissipation is present. The related cooling time *t*_8/5_ is calculated according to Eq. 3. It can be seen that *t*_8/5_ increases with the square of heat-input $Q$ [kJ/mm] and is inversely proportional to the square of workpiece thickness *d* [mm]. The calculation of *t*_8/5_ for the low alloy steels with small thicknesses is simplified according to Eq. 4.

3 $${t}_{8/5}=\frac{{Q}^{2}}{4\pi \lambda \varrho c{d}^{2}}\times \left[{\left(\frac{1}{500-{T}_{0}}\right)}^{2}-{\left(\frac{1}{800-{T}_{0}}\right)}^{2}\right]\times {F}_{2}$$

4 $${t}_{8/5}=(4300-4.3{T}_{0})\times {10}^{5}\times \frac{{Q}^{2}}{{d}^{2}}\times \left[{\left(\frac{1}{500-{T}_{0}}\right)}^{2}-{\left(\frac{1}{800-{T}_{0}}\right)}^{2}\right]\times {F}_{2}$$

where ${T}_{0}$ [°C] is the preheating temperature and $Q$ is calculated according to Eq. 5. *F*_2_ (= 1 for bead-on-plate weld seam) is the seam factor for two-dimensional heat dissipation. The parameters $\lambda$ [J/(cm⋅K⋅s)], $\varrho$ [kg/m^3^], and $c$ [J/(kg⋅K)] are material properties, i.e. thermal conductivity, mass density, and specific heat capacity, respectively.

5 $$Q=\eta UI/(1000v)$$

where $\eta$ is the thermal efficiency and an empirical value of 0.65 is selected for the bead-on-plate TIG welding process (pure Ar as the shield gas) with DCEN. $U$ [V] and $I$ [A] are the welding voltage and welding current, respectively. $v$ [mm/s] is the moving speed (or welding speed) of the TIG torch.

Thermal-physical properties of $\lambda$, $\rho$, and $c$ of S700MC steel (hot-rolled according to EN 10149–1 and EN 10149–2) were calculated using the JMatPro 13.3 software. The calculations were based on the “general steel” database for a temperature range of [25 °C, 1200 °C] and then fitted polynomially as functions of temperature *T* [°C]. The calculated data and fitting curves were plotted in Fig. 15. High ${R}^{2}$ of each fitting curve indicates the good-fitting performance.

## Appendix 3

The Welding CCT (WCCT) diagrams for the S700MC steel were studied from the researches [23, 24]. Two WCCT diagrams of S700MC steel were established at two peak heating temperatures of 1000 °C at the heating rate of 340 °C/s and 1300 °C at the heating rate of 575 °C/s. The microstructure evolutions and Vickers hardness (HV 10) of S700MC steel to the cooling time *t*_8/5_ [s] were modelled exponentially according to Eq. 6 by nonlinear least squares fitting method. Besides, the martensite starting temperature (Ms °C) was fitted linearly by the least-square method (see Fig. 16 (e)).

6 $$y={\text{a}}*{\text{exp}}\left({\text{b}}*{t}_{8/5}\right)+{\text{c}}*{\text{exp}}({\text{d}}*{t}_{8/5})$$

Figure 16 shows those fitting curves; a high *R*^2^ for each curve indicates the good goodness of fitting. The related coefficients were summarised in Table

**Table 7:** Coefficients of exponential model for modelling microstructures of the S700MC steel versus the cooling time t_8/5_ [s] for two peak heating temperatures

Peak T [°C]	Heating rate [°C/s]	Compositions and hardness	Coefficients of exponential model with two terms (Eq. 3) [-]			
			a	b	c	d
1000	340	Martensite	30.50627	− 0.40935	0.0279	0.02732
		Bainite	1.19868E + 3	− 0.02418	− 1.12599E + 3	− 0.02351
		Retained austenite	3.13277	− 0.01402	3.77022E-5	0.09325
		Ferrite	75.5613	0.00267	− 71.6111	− 0.07389
		Hardness [HV 10]	56.7659	− 0.0456	189.4760	− 0.0005
1300	575	Martensite	4.14933E + 5	− 0.3386	− 4.14855E + 5	− 0.3387
		Bainite	104.5225	− 0.00131	− 107.8789	− 0.14298
		Retained austenite	6.1534	− 0.00429	− 5.1871	− 0.04254
		Ferrite	23.8637	0.00966	− 23.94765	0.0093
		Hardness [HV 10]	101.62299	− 0.12515	223.42316	− 5.29681E-4

^7^ (without displaying the units). These fitting models could predict the continuous evolutions of microstructure and hardness of the S700MC steel versus the cooling time *t*_8/5_.

In addition, the microstructures of FZ at the calculated *t*_8/5_ of 5.4 s, 16.7 s, and 26.5 s were calculated and plotted in Fig. 17 a, b, and c, respectively. The microstructures were calculated from the solidification prosperity of S700MC steel using JMatPro 13.3 software at a given cooling rate and grain size and described by phase fractions.
